# Applications of Curcumin and Its Nanoforms in the Treatment of Cancer

**DOI:** 10.3390/pharmaceutics15092223

**Published:** 2023-08-29

**Authors:** Deepa Mundekkad, William C. Cho

**Affiliations:** 1 Department of Biotechnology, Nehru Arts and Science College, Thirumalayampalayam, Coimbatore 641105, India; 2 Department of Clinical Oncology, Queen Elizabeth Hospital, Hong Kong, China

**Keywords:** curcumin, nanocurcumin, anti-cancer, immunomodulation, signaling pathways, immune tolerance, co-stimulation, turmeric, immune checkpoint

## Abstract

Due to the diverse medicinal and pharmacokinetic properties of turmeric, it is well-known in the therapeutic, pharmaceutic, nutraceutical, cosmetic, and dietary industries. It gained importance due to its multitude of properties, such as wound-healing, anti-inflammatory, anti-oxidant, anti-microbial, cytoprotective, anti-aging, anti-cancer, and immunomodulatory effects. Even though the natural healing effect of turmeric has been known to Indians as early as 2500 BCE, the global demand for turmeric has increased only recently. A major reason for the beneficiary activities of turmeric is the presence of the yellow-colored polyphenolic compound called curcumin. Many studies have been carried out on the various properties of curcumin and its derivatives. Despite its low bioavailability, curcumin has been effectively used for the treatment of many diseases, such as cardiovascular and neurological diseases, diabetes, arthritis, and cancer. The advent of nanobiotechnology has further opened wide opportunities to explore and expand the use of curcumin in the medical field. Nanoformulations using curcumin and its derivatives helped to design new treatment modalities, specifically in cancer, because of the better bioavailability and solubility of nanocurcumin when compared to natural curcumin. This review deals with the various applications of curcumin nanoparticles in cancer therapy and broadly tries to understand how it affect the immunological status of the cancer cell.

## 1. Introduction

**Figure 1:**
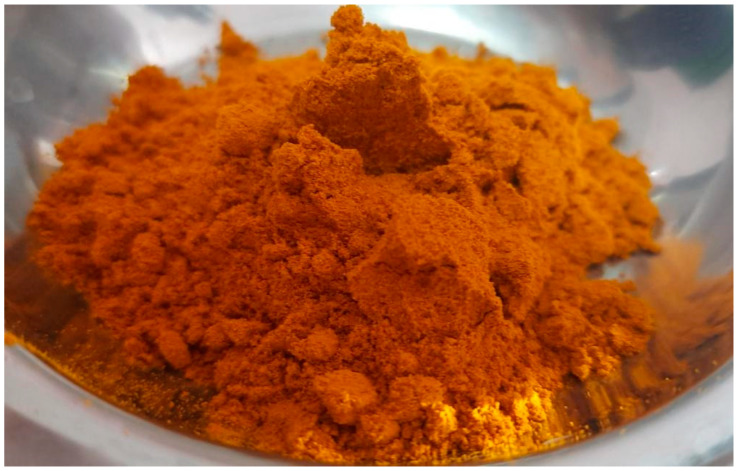
Turmeric powder. The rhizomes of *Curcumin longa* are harvested, cooked, dried, and powdered to obtain the aromatic yellow turmeric powder.

Turmeric, the yellow powder obtained from the rhizome of *Curcuma longa,* is an important spice used extensively in India and China. Turmeric is well-known for its medicinal, therapeutic, nutraceutical, and dietary usage [1]. As per Ayurveda, the indigenous Indian medical system, turmeric is used for a multitude of purposes. In cuisine, turmeric is used to enhance color and flavor. A majority of Indian cuisine use turmeric as a base ingredient due to the healing properties inherent in the polyphenol-rich yellow powder (Figure 1). Turmeric is well-known for having curative properties [2]. Indians, from time immemorial, were known to consume turmeric from early childhood giving them relief from dyspepsia, flatulence, indigestion, and acidity, usually happening after taking spicy food. It could be postulated that this property of turmeric could have contributed to the tolerance level of Indians to very spicy food. Some of the indigenous practices and the curative effects of turmeric practiced in India since ancient times are enlisted in Table 1.

**Figure 2:**
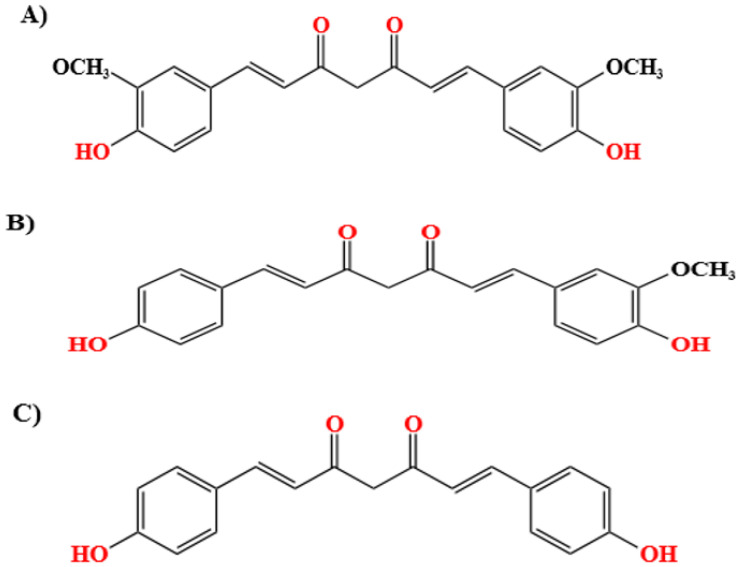
Chemical structure of curcuminoids. (**A**) curcumin, (**B**) demethoxycurcumin and (**C**) bis-demethoxycurcumin.

Currently, turmeric has a global market as the therapeutic potential of curcumin, the main component of turmeric, attracted a lot of attention among researchers, scientists, and medical practitioners due to the increased awareness of the anti-inflammatory, anti-diabetic, anti-cancer, and anti-aging properties of curcumin. The pharmaceutical and cosmetic industries also profoundly benefited from the pharmacokinetic property of curcumin [8]. Turmeric contains two more important analogs of curcumin-demethoxycurcumin and bis-demethoxycurcumin (Figure 2), together called curcuminoids, which are responsible for most of the properties attributed to turmeric. Curcuminoids are hydrophobic and insoluble; it is well known that normal curcumin is not available for systemic circulation due to its insoluble nature. However, when they are in nanoform, the gut bacteria can metabolize them more effectively to active metabolites such as tetrahydrocurcumin. The specific microbe present in the gut, *E. coli*, converts curcumin first to the intermediate dihydrocurcumin, which will later be converted to tetrahydrocurcumin by a mechanism called reductive destruction [9]. Another strain of bacteria, *Blautia* sp. (MRG-PMF1), uses a demethylation reaction to convert curcumin to demethylcurcumin and bisdemethylcurcumin. These derivatives of curcumin are readily available for systemic circulation [10]. Instead of the portal vein, if curcumin and its nanoforms are circulated through the lymphatic system, they can be absorbed more effectively and, as a result, can directly act as an anti-inflammatory agent by binding to Toll-like receptors (TLRs). TLRs have an important role in mediating innate immunity and are implicated in the pathogenesis of chronic inflammatory diseases. The anti-inflammatory activity can also be due to the down-regulation of the signaling components (such as the Nuclear factor kappa B (NF-κB), Mitogen-Activated Protein Kinases (MAPK), or Activator Protein 1 (AP-1)) of the inflammatory pathway. Thus, it is clear that curcumin metabolites can regulate inflammatory mediators and, thus, treat inflammatory diseases [11,12].

The wound-healing property of curcumin is the most studied significant medicinal quality that can be attributed to the antioxidant and anti-inflammatory properties of curcumin. In traditional medicine, the inflammation associated with wounds is found to be reduced by the topical application of turmeric on wounds [13], confirming the anti-inflammatory activity of curcumin. The ability of curcumin to control the influx of histamine and inflammatory mediators, such as platelet-derived growth factor (PDGF), fibroblast growth factor-2 (FGF-2), tumor necrosis factor-α (TNF-α), and interleukin-1 (IL-1), is the main reason for the wound-healing and anti-inflammatory properties. However, many of these inflammatory mediators are found to be closely associated with other disease conditions such as arthritis, metabolic syndrome, obesity, neurodegenerative diseases, etc. [8]. Incidentally, some of these conditions are also a prelude to cancer, and many types of cancers (such as liver, cervical, and inflammatory breast cancer) are found to show signs of immunodeficiency, inflammation, and metabolic disturbances. Cancer being an umbrella term, is not a single disease but a myriad of 200 or more diseases. With every newly grown cancer cell, there is a chance of having a new mutation. This mutation during the development of cancer causes problems during treatment as mutations can also bring about chemoresistance to the cells. The occurrence of multiple conditions in a single disease makes it difficult to control the disease, as targeting multiple disease conditions will require different treatment modalities.

Many of the medicinal herbs that are mentioned in the ancient literature and texts possess different activities that are present in the same plant. For example, *Aegle marmelos*, commonly known as Bael, is found abundantly in the Indian Subcontinent. It is known to have digestive, anti-inflammatory, anti-ulcerative, wound-healing, and anti-dote properties [14]. Turmeric is another medicinal herb where the main component, curcumin, offers solutions for many complications, such as inflammation, diabetes, and cancer, due to its various biological properties. The therapeutic potential of curcumin, evidenced by pre-clinical and clinical studies, revealed its ability to cure a variety of conditions, such as rheumatoid arthritis, migraine, polycystic ovarian syndrome, premenstrual syndrome, nonalcoholic fatty liver disease, knee osteoarthritis, psoriasis, atherosclerosis, amyotrophic lateral sclerosis, liver cirrhosis, ulcerative colitis, depression, and Alzheimer’s disease [15].

Cancer is the major cause of death worldwide. Cancer cells tend to proliferate unconditionally in the tumor microenvironment. This unconditional growth of cancer cells may be due to the resistance that the cancer cells have developed against the conventional medicines that are used these days. The resistance strengthens the immune system of the cancer cells and can, in turn, result in the decreased effectiveness of the drugs used [16]. Cancer cells become tolerant to the medicines and develop resistance. In patients who have undergone preliminary treatment for cancer, relapse and drug resistance can also happen due to the presence of cancer cell clones. Understanding the seriousness of the situation, scientists are in search of remedies for different types of cancers that are resistant to the current treatment modalities. The recent development of nanoparticles (NPs) as anti-tumor agents has opened up new avenues to explore in the field of nanobiotechnology and nanomedicine.

**Figure 3:**
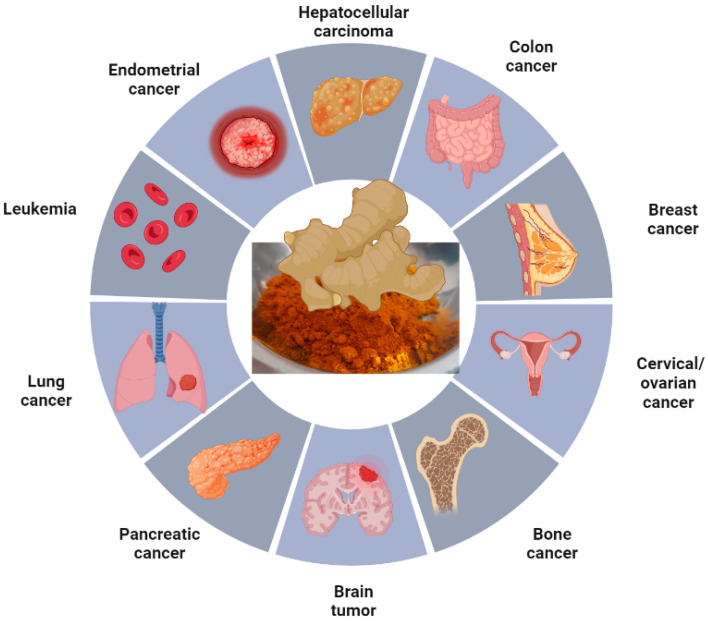
Various types of cancer, which are being treated using curcumin and its nanoforms. (Figure prepared using BioRender.com).

**Figure 4:**
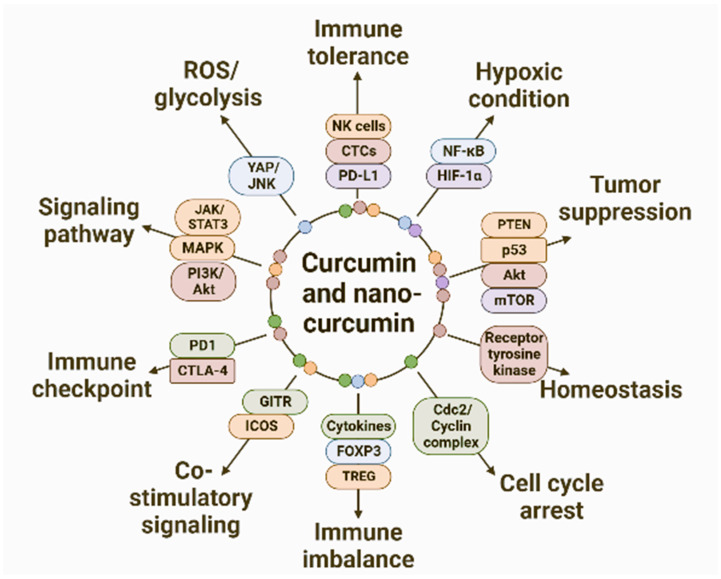
Some of the molecular events and the corresponding molecules in cancer, which are affected by the treatment with curcumin and its nanoform. (NK cells—natural killer cells; CTCs—circulating tumor cells; PD-L1—programmed-death ligand 1; NF-κB—nuclear factor-kB; HIF-1α—hypoxia inducing factor-1α; PTEN—Phosphatase and Tensin Homolog; p53—tumor suppressor protein; Akt—protein kinase B; mTOR—mammalian Target Of Rapamycin; Cdc2—conventional dendritic cell type 2; FOXP3—forkhead box P3; TREG—Regulatory T cells; GITR—Glucocorticoid-induced TNF receptor; ICOS—inducible T-cell co-stimulator; PD1—programmed death 1; CTLA-4—cytotoxic T-lymphocyte associated protein 4; JAK—Janus Kinase; STAT3—signal transducer and activator of transcription; MAPK—Mitogen-activated Protein Kinase; PI3K—Phosphatidylinositol-3-kinase; YAP—Yes-associated protein; JNK—c-Jun N-terminal kinase). (Figure prepared using BioRender.com).

Curcumin and its nanoforms have anti-cancer activity against various cancer types (Figure 3). Curcumin influences various signaling pathways, thus regulating the tumor microenvironment and, thereby, tumor progression. Some of the molecular events and the related molecules that are affected by curcumin and its nanoform are represented in Figure 4. The activities of these molecules are regulated by such mechanisms as cell death, cell cycle progression, protein kinase activity, and expression of transcription factors and other proteins such as Trop2. When human oral cancer SAS cells were treated with γ-polyglutamic acid (γ-PGA)-coated NPs that were loaded with gefitinib/curcumin (Gef/cur), the cells internalized the γ-PGA-Gef-Cur NPs. The curcumin-loaded NPs induced caspase- and mitochondria-mediated cell death in the cancer cells [17]. The chitosan-poly(lactic-co-glycolic acid) (PLGA) NPs loaded with curcumin were capable of penetrating the blood–brain barrier when modified with sialic acid (SA) and anti-aldehyde dehydrogenase (anti-ADH). These NPs were used for targeting brain cancer stem cells (BCSC) and glioblastoma U87MG cells. This complex inhibited the growth of BCSC and U87MG cells when ADH was targeted; ADH was co-localized with CD44 in both cell lines. The chitosan-PLGA-curcumin NP complex was further developed in such a way as to release and deliver the curcumin to brain cells to inhibit the growth of glioblastoma and BCSC cells [18]. Similarly, a nanoformulation based on poly-glycerol-malic acid-dodecanedioic acid (PGMD)/curcumin nanoparticle was developed to test the efficacy of breast cancer cell lines (MCF-7 and MDA MB-231). Apart from growth inhibition, nuclear anomalies and other apoptotic features were also observed in the cells treated with the curcumin nanocomplex. Apoptosis induction was confirmed by assays measuring the amount of caspase-9 in treated cells [19]. The cytotoxicity of curcumin was improved by the co-delivery of Dox with graphene as the nanocarrier. The synergistic effect of Dox and nanocurcumin resulted in high drug-loading efficiency with localized delivery and controlled release of curcumin in the cells tested—AGS, PC3, MCF-7, and HFF [20].

The use of nanoparticles has become the standard approach for the treatment of cancer through various modes such as targeted drug delivery, conjugation of nanoparticles with conventional drugs, surface modification of nanoparticles by prodrug (such as a polymer backbone) conjugation, etc. [21]. The therapeutic capacity of nanoparticles is mainly due to their flexibility—their biocompatibility, stability, selective binding with the tumor receptors on the cell surface, etc., that make them ideal as anti-tumor agents [22]. Nanoparticles are also found to influence various signaling pathways that control the molecular events leading to cell proliferation and differentiation, thus controlling cancer progression. Although the hydrophobic nature of curcumin makes it difficult to cross the membrane, the nanoforms of curcumin can offer better penetrating power, thus increasing bioavailability [23]. Limitations of curcumin, such as the systemic elimination (fast clearance of curcumin from the body) and poor water solubility, can be managed by using nanoforms of curcumin [24] where the drugs can be encapsulated in a suitable nanoform and can be delivered appropriately. This review tries to focus on novel aspects, such as the influence of nanocurcumin on cancer and the various pathways that are influenced as a result of the treatment with curcumin and its various nanoforms.

As discussed, nanoparticles conjugated with conventional drugs can have additional curative effects. Nanocurcumin has evolved as a promising therapeutic modality in cancer treatment with enhanced and efficient immunomodulatory effects [25]. The number of clinical trials with curcumin as the main compound or as an adjuvant is evidence of its efficiency in controlling tumor growth (Table 2). Nanocurcumin is found to have a targeted cytotoxic effect on the leukemic stem cells that act as cell clones by inducing apoptosis [26]. This is because conjugating nanocurcumin with antibodies improves bioavailability and helps in the directed delivery of the nanocurcumin to the tumor site. Nanocurcumin also influences the cellular signal transduction pathways that have an important role in tumor transformation by suppressing and modulating the signaling molecules along with the modulation of apoptotic regulators such as p53, Bax, and caspases.

Another concern in cancer therapy is the unselective targeting of tissues by conventional drugs resulting in relapse and chemoresistance [27]. Resistance to multiple drugs is common in cancer, especially in triple-negative breast cancer (TNBC). This resistance is offered by drug efflux transporters (such as P-glycoprotein transporter) associated with ATP (Adenosine Triphosphate)-binding cassettes (ABC). Curcumin is known to have inhibitory effects on such transporters. The toxicity of doxorubicin (Dox) was increased 10-fold in TNBC by conjugating with curcumin loaded with solid lipid nanoparticles. The increase in toxicity was attributed to the association of curcumin nanoparticles that reduced the oxidative stress in the cells, thereby affecting the transcriptional activation of the P-glycoprotein transporter mediated by p65/p50 NF-κB [28]. The inhibition of the transcriptional activation of NF-κB results in the failure of its translocation to the nucleus. The transcription of proteins further downstream the pathway is affected, and it appears that the inhibition of the transcriptional activity of NF-κB negatively affects the expression of oncogenic and inflammatory proteins as well. Many of the signaling pathways are affected by the end result that curcumin can act as an anti-proliferative, anti-inflammatory, antioxidant, and anti-cancerous molecule [29].

As such, tumors are heterogeneous, and it is essential to understand the various molecular mechanisms that lead to the cytotoxic effects influenced by nanocurcumin. This is intended to impact and encourage further research and developmental activities resulting in the discovery of new molecular targets [30], which offers therapeutic effects in various types of cancers. Even though the review is inspired by research dealing with the anti-cancer effects of curcumin, a detailed study on the immunomodulatory aspects of curcumin and its nanoform is also attempted. It is expected that this review will help to understand the potential applications of curcumin and its nanoforms in various aspects of cancer therapy, especially immunotherapy, where emphasis is given to the role of curcumin in immunomodulation and various related events such as immune tolerance, co-stimulation, and co-inhibition. Description of the activation of immune responses mediated by a secondary signal called co-stimulatory signal to activate immune responses in the presence of the antigen-presenting cells (APCs) also appears in the respective sections. In the future, the researcher may explore various other molecular targets, such as biomarker-integrated approaches for cancer therapy, using this information.

## 2. Methodology Used

**Figure 5:**
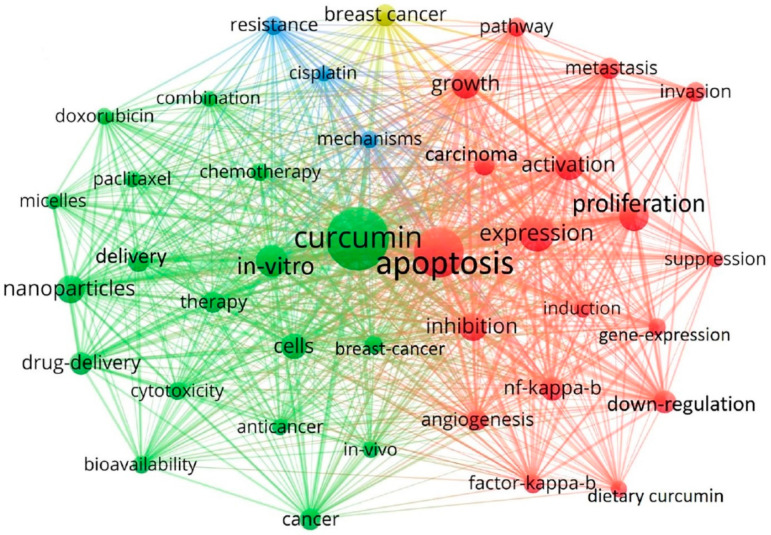
A keyword co-occurrence map reflecting the research hotspot in the field. (Adapted from Zhang et al., 2022 [31], with minor change). The figure created using VOSviewer software (VOSviewer version 1.6.18) for visual analysis reflected the keywords that appeared in publications in recent years, and indicated some of the terms that are currently the hot or cutting-edge research topics in these fields related to curcumin and tumors. Green color represents the keywords that are from the most recent publications, followed by keywords represented in red color. Blue-colored keywords appeared in articles prior to yellow-colored keywords. The larger the size of the circle, the more occurring the keyword is in the articles.

A systematic search of the PubMed database and Google Scholar was conducted to obtain the results for studies related to the effect of curcumin and nanocurcumin on various types of cancers. The search algorithm used was cancer AND nanocurcumin AND anti-cancer AND cancer therapy AND nanocarriers AND drug target AND immunomodulation [31]. The Preferred Repeated Items for Systematic Reviews and Meta-Analyses (PRISMA) guidelines and checklist were followed for the systematic search. Inclusion criteria were decided based on the Problem/Population, Intervention, Comparison, and Outcome (PICO) terms. Published studies from the year 2000 onwards were considered, with preferential emphasis on the major studies conducted in the past five years (2019–2023)—96 studies from a total of 138 are from the past five years. This means that around 70% of the references were taken from the past five years. References older than five years are considered only when a statement is important in the context and needs to be emphasized. An overview of inclusion and exclusion criteria is given below in Table 3. To emphasize the keywords used for the systematic search reflecting the hotspot topics in the field, a keyword co-occurrence map is also provided (Figure 5).

## 3. Curcumin and Its Properties

Curcumin can be considered a neutral compound in terms of pharmacological and toxicological effects. It has minimum toxicity when administered to animals or humans, even at relatively higher doses as compared to other traditional drugs. As already discussed, curcumin occurs in different isomeric forms, and due to this, there is a wide scope for the application of curcumin in the fields of pharmacology and medicine. Curcumin has three different p*K*_a_ values (the first and second p*K*_a_ values are contributed by the phenolic hydroxy groups and the third one by the enolic proton). With three different p*K*_a_ values, the dissociation of protons is easy. The proton-transfer-dissociation is closely associated with a radical scavenging mechanism [32], and thus, curcumin has a wide radical-scavenging activity [33]. Recently, curcumin was classified as belonging to the category of PAINS (pan-assay interference compound). PAINS predominantly mediate non-covalent interactions along with different levels of binding affinity. Because of this vast option in binding energy, curcumin and its nanoforms have a range of applications in medicine. However, despite the extensive range of applications, there are some limitations, as discussed below.

### 3.1. Bioavailability

The low bioavailability is always an issue for the biological applications of curcumin. Curcumin is rapidly metabolized and excreted from the body soon after administration, and because of this, the availability and clinical scope become limited. This is partially due to the conjugation reactions, such as sulfation and glucuronidation, that curcumin undergoes at different tissue sites when administered orally to the body [34]. The rate of metabolism is so fast—almost 90% of the supplied curcumin is metabolized in 30 min. The breakdown and absorption of curcumin can be delayed by encapsulating it in a suitable nanoform, such as liposome or micelle, and making it resistant to early digestion by the gut bacteria. This way, the bioavailability could be increased over a nine-fold [35,36]. Furthermore, it was found that the binding efficiency of nanocurcumin favors hydrophobic interactions, thus increasing the bioavailability with up to a 60-fold increase in half-life, making its use in cancer therapy even more promising [37]. The low bioavailability could be due to issues with absorption or distribution.

#### 3.1.1. Absorption

Oral dosing of any drug typically depends on its absorption capacity. Curcumin has poor permeability across membranes, and therefore, the absorption of curcumin is very negligible, as evidenced by several studies [34,38,39,40]. Upon oral administration, very low concentrations of curcumin were observed in the blood plasma of rats. However, recently, studies have revealed that nanoformulations could increase bioavailability and, thus, increase therapeutic efficiency without any possible side effects. Liposomes [41], micelles [42], nanoparticles [43,44], nanogels [45], nanocrystals [46], etc., are known to increase the availability and absorption capacity of curcumin. The route of administration also defines the rate of absorption. For example, the intraperitoneal route revealed more curcumin in plasma than either intravenous or oral routes of administration.

#### 3.1.2. Distribution

Biological activity is highly dependent on the distribution of curcumin in the body tissues, especially in the liver and kidney. In this case, the amount of curcumin found in various tissues is dependent on the route of administration (intraperitoneal or gavage or dietary intake). The highly reactive structure of curcumin leads to its easy degradation even before it is properly absorbed, resulting in poor distribution to specific locations [47]. When cancer patients were injected with liposomal curcumin, the pharmacokinetic parameters were found to be greatly changed. The plasma concentration showed that when disposed of in red blood cells, the level was elevated to a greater extent with a shorter elimination phase [48].

### 3.2. Physical and Chemical Properties of Curcumin

Curcumin exists as a keto-form in acidic and neutral pH, whereas in alkaline pH, it exists predominantly in the enol-form. The anti-oxidant radical scavenging activities of curcumin are majorly contributed by the enol-form, which can donate electrons. The curcumin structure is stabilized by the resonance-assisted hydrogen bonding in the enol-form, where it undergoes Excited State Intramolecular Hydrogen Transfer (ESIHT). ESIHT enhances catalytic and biological activities, which explain the various reactive and biological activities of curcumin [49]. Further to this, the anti-inflammatory activity is endorsed by the presence of two phenolic groups separated by a hydrophobic bridge in an enolic center. Nanocurcumin can easily penetrate the blood–brain barrier facilitated by the hydrophobic and hydrophilic interactions. In addition, the distribution, uptake, and circulation of curcumin are affected by molecular size. The nanosize of compounds is a huge determinant of uptake and circulation. As compared to normal curcumin, nanocurcumin with a smaller size have a better half-life period, uptake capacity, and improved capacity for drug delivery [50].

### 3.3. Anti-Cancer Activity of Curcumin

The ability of curcumin and curcumin-related nanoformulations to suppress the proliferation of cells has helped in its extensive use in the treatment of cancer. This ability is due to the presence of a wide variety of polyphenolic compounds in curcumin that has a suppressive effect on the growth of cells. These effects are mediated through the transcriptional regulation of tumor suppressor/promoter genes. Research showed that curcumin is capable of controlling most of the transcription factors, cytokines, cell adhesion molecules, growth factors, and their receptors, emphasizing its role in controlling the initiation, progression, and metastasis of tumors. It is also found to be acting by directly binding to tubulin and reducing GTPase activity, thereby exerting its inhibitory action on the tubulin polymerization [51]. The other major mechanism of action is the control of reactive oxygen species (ROS) through anti-oxidant activity along with the control of prostaglandins (such as prostaglandin E2-PGE2), cyclooxygenase-2 (COX-2), tumor necrosis factor-α (TNF-α), interleukins, etc. Different types of cancers, such as breast, prostate, pancreatic, colorectal, etc., were also studied to understand the effect of curcumin, and many pilot, clinical studies were successfully conducted to detect and establish the therapeutic effects of curcumin and its various formulations [52]. Transcription factor (such as NF-κB) is another important molecule that is affected by curcumin treatment. The various pathways upstream and downstream of this important protein complex are modulated by treatment with curcumin in its original and nanoforms. The inhibitory effects that curcumin has on NF-κB and Wnt/β-catenin pathways explain the ability of curcumin to impair cancer cell growth.

The anti-proliferative effect of curcumin is one of the most popular researched topics. Almost all types of cancers are studied, and curcumin was found to be effective in controlling cancer cell growth in most of the tumor types studied in vitro. The anti-proliferative effect can be taken as an extension of curcumin’s biological activities. The multi-dimensional effect of curcumin (effect on signaling pathways, immunomodulation, cell growth, etc.) also contributes to the anti-proliferative effect of curcumin. If utilized properly with clinical support, the anti-proliferative activity can be explored further to develop a clinically approved formulation using curcumin. Since there is no such FDA-approved curcumin derivative available in the market, there is a huge scope for developing one.

## 4. Nanoforms of Curcumin

### 4.1. Technologies Used to Fabricate Nanocurcumin

Nanoforms of curcumin are prepared by various technologies. A solution-enhanced dispersion of curcumin nanoparticles was prepared by supercritical carbon dioxide. The method, called SEDS, was designed to determine the influence of the size of the nanocurcumin on various optimal parameters such as rate of flow or temperature. The significance of size on these factors was determined by this analysis [53]. Another novel method employed flash nanoprecipitation for the preparation of curcumin nanoparticles using a coarse oil-in-water emulsion. This method involved a turbulent co-mixing of water with emulsion-carrying curcumin using a manually-operated confined impingement jets mixer. A very clear and stable dispersion of curcumin nanoparticles was formed by this process [54]. There is the possibility of converting this to dry, easily water-dispersible powder by spray drying, making storing and application much easier than for emulsion.

Coacervation is a technique where a polymer is dissolved in an organic solvent, and here, curcumin is directly suspended in this polymeric solution to homogenize and form the nanocurcumin. The nanoparticles can be collected by centrifugation after the process. When the mixture of polymer and curcumin is continuously stirred in water, precipitation occurs, and this is another procedure to form nanocurcumin. Nanoforms of curcumin can also be formed by the spray drying method, where a simple spray dryer is used to receive nanocrystals. The solvent can be evaporated from the solution to obtain the nanoparticles. The thin film hydration method is another mode of curcumin nanoparticle preparation where curcumin mixed with a suitable surfactant is sonicated and centrifuged to obtain the nanoparticles.

### 4.2. Activities of Curcumin Nanoforms

The nanoforms of curcumin have better functional ability than natural curcumin due to the enhanced pharmacokinetic activities. The nanoencapsulated curcumin has better bioavailability and facilitates accelerated metabolism. The efficiency of transport of the drug to the area of action is increased when the nanoform of curcumin is used as the delivery agent. Polymeric nanoparticles, nanofibers, nanoemulsions, liposomes, and various other forms of curcumin nanoparticles are used as such or in combination with other drugs to increase efficiency. In addition to the metabolic efficiency, the therapeutic potential of curcumin is increased upon its configuration to nanoform. The antimicrobial activity is found to be superior when in the nanoform [55]. The antibacterial activity of curcumin nanocomposites was found to be stimulated due to synergistic energy transfer from silver nanoparticles to the curcumin [56]. Extensive reviews are reported on the biological activities of curcumin and its nanoforms against such pathogens as bacteria, viruses, fungi, etc. [57]. The activity against protozoal and other parasitic lifeforms is also explored in detail in these studies. Photoexcitation is found to have an effect on the increased activity. The bacterial tubulin homolog FtsZ protein is vital in cell division initiation. The antimicrobial activity of curcumin nanoparticles is mostly related to the activity of this protein [57]. Apart from the modulation of free radical production and destruction, controlling the enzymatic ROS-initiators, such as cyclooxygenase and lipoxygenase, contributes to the antioxidant activity of the nanocurcumin [58]. Various signaling pathways, such as NF-kB, are involved in the anticancer activity of the nanocurcumin, whereas inflammatory mediators guide the anti-inflammatory and pro-inflammatory activities [59,60].

## 5. Types of Nanocarriers Used for Curcumin Delivery

**Figure 6:**
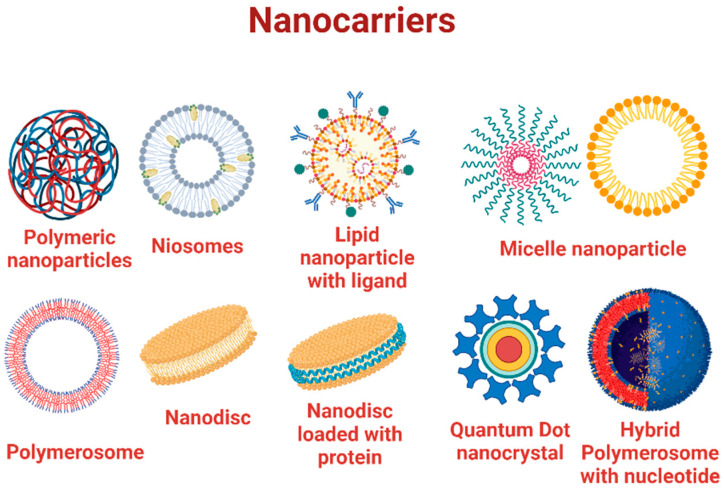
Various nanocarriers, which act as vehicles for delivery of drug molecules.

Nanocarriers (Figure 6) are designed to facilitate the easy transport of drugs, especially those that are not naturally carried across the membrane. Curcumin nanoparticles, functionalized with polymeric, metallic, or lipidic nanoparticles, can easily be carried across the membrane and released at the target. Curcumin loaded with biocompatible polyester-origin polymer poly(D,L-lactide), PLA, was used to study the stabilization and cytotoxicity, where the encapsulated nanocurcumin showed higher resistance to photobleaching as well as photodegradation [61]. Microscopic lamellar structures called niosomes can be loaded with metal nanoparticles, along with curcumin resulting in an enhanced drug delivery [62,63]. This is a great addition to cancer therapy, as photobleaching and photodegradation are challenges encountered in photodynamic adjuvant therapy in cancer. Furthermore, a radical change is observed in the internalization potential of curcumin when it is functionalized with moieties that can interact with biological molecules [64]. Curcumin functionalized with gold nanoparticles was tested for cytotoxicity in the human prostate cancer cell line. The curcumin nanoparticle was found to induce increased cytotoxicity in the cell line tested. The cellular uptake and the subsequent cytotoxicity were guided by the surface chemistry and solubility of curcumin nanoparticles emphasizing the drastic increase in toxicity upon functionalization with gold nanoparticles. Curcumin-cyclodextrin/cellulose complexes (CNCx) were developed to serve as a drug delivery system where the functionalization by ionic association resulted in anti-proliferative effect when tested in the prostate (PC-3 and DU-145) and colorectal (HT-29) cancer cell lines. The enhanced permeability and retention (EPR) effect and solubility were postulated to be the reasons behind the anti-proliferative effect of the nanocomplexed curcumin compared to the bare curcumin [65]. Liposome nanoparticles loaded with curcumin synthesized by the electrospray process were successfully employed for the delivery of drug tested in a kinetically controlled environment. The electrostatic interaction between the core polymeric compound and the drug molecules created repulsive forces inducing the easy release of drug molecules to the target site. The presence of functional groups on the surface of the functionalized curcumin resulted in increased drug release due to enhanced solubility through intermolecular hydrogen bonding. More curcumin is released, resulting in an enhanced cytotoxicity as observed in HeLa cells [66]. The controlled release of curcumin from thiolated starch-coated iron oxide magnetic nanoparticles resulted in cytotoxicity in MCF-7 and HepG2 cell lines. The drug loading and encapsulation efficiency was enhanced with more polymer coating, effectively imparting an anti-proliferative effect to the nanoparticles [67]. Internalization was improved when curcumin and Dox were incorporated into the PEGylated liposomes. This nanoformulation was found to have enhanced cytotoxicity. The expression levels of phosphorylated NF-κB p65 were found to be decreased in the cell lysate of C26 cells treated with the liposomal curcumin nanoformulation. Angiogenic and inflammatory mediators were also negatively regulated by the formulation, where the effect was specifically attributed to the presence of curcumin in the nanoform [68].

Due to the hydrophobic nature of curcumin, it is not soluble in water in normal conditions. On the one hand, this property can be used to design curcumin as a carrier molecule for soluble compounds that are normally degraded immediately after administration. On the other hand, when incorporated with polymeric micelles, and liposomes, curcumin can be easily transported across the membrane to offer better solubility and therapeutic effect. In such cases, it is important to see that the carriers of curcumin are compatible with the tumor cells and does not offer unwanted toxic effect.

The different carrier systems for the efficient encapsulation of curcumin are briefly discussed in the following section.

### 5.1. Protein-Based Encapsulation of Curcumin for Delivery

Bio-polymers based on protein were used to encapsulate curcumin and deliver it through various routes. Albumin is one such protein that offers highly variable binding sites for the conjugating molecule. Curcumin encapsulated in albumin has higher stability and bioavailability and, being non-immunogenic, has fairly good compatibility with the immune system. Bovine serum albumin (BSA) and Human serum albumin (HSA), are the most commonly used proteins for encapsulation [69,70]. Zein, a protein obtained from the kernels of corn, is also a likely candidate drug carrier due to the presence of non-polar amino acids. It is used as a coating where the accessibility and bioavailability of curcumin is increased considerably. Encapsulation with zein protein was also found to prevent the degradation of curcumin. The other benefit of using zein is that the encapsulated curcumin becomes heat tolerant, leading to a better bio-availability [71]. The insoluble silk fibroin is also used for the encapsulation and delivery of curcumin nanoparticles, where the mechanical tensile strength and flexibility of the fibroin aid in the effective delivery of curcumin [72].

### 5.2. Polysaccharide-Based Encapsulation of Curcumin for Delivery

The glycosidic linkages in polysaccharides favor the formation of branched molecules giving the flexibility to conjugate with other molecular forms. The efficiency of polysaccharides to encapsulate and deliver the target drug to the site of action encouraged the extensive use of polymers, such as chitosan [73], alginate [74], cellulose [75], and starch, [76] in various curcumin nanoformulations. Polysaccharide encapsulation ensures stability in terms of storage and response to varying pH [77].

### 5.3. Self-Assembled Molecules and Ligands as Carriers for Delivery of Curcumin

The ability of the carrier molecule to modulate the physical and chemical properties to carry the drug more efficiently is considered to be an important aspect of drug delivery. Certain features, such as crystallinity, versatility, modularity, porosity, biocompatibility, etc., play an important role in deciding the drug-carrying efficiency of the carrier molecule.

#### 5.3.1. Metal–Organic Framework (MOF)

Crystalline, porous materials called MOF are used for carrying drug molecules across the cell system. MOFs are found to be highly promising in the biomedical field, especially in targeted delivery. MOFs can overcome the limitations faced by conventional drug delivery systems, such as stability, drug-carrying capacity, bioavailability, solubility, etc. MOFs contain metal ions that are interconnected by organic ligands. The ligands or linkers are labile, have large surface areas, and are relatively less toxic for the cells when used biologically. MOFs were found to incorporate curcumin and studied as an anticancer drug [78].

#### 5.3.2. Covalent Organic Frameworks (COFs)

These molecules are specific in that they can assemble themselves to form crystalline materials called covalent organic frameworks (COFs), forming the newest trend in biomedical therapeutics. The advantage of these COFs is that the porosity allows for more options for drug loading in their porous channels. The pore size and porosity of these materials can be regulated. COFs are more functional and versatile in terms of porosity, compatibility with biological cells, and efficient loading capacity [79]. A self-assembled nanocomposite COF with curcumin was efficiently loaded and used as a carrier of conventional drugs, such as Doxorubicin, providing excellent cell affinity due to an improved dispersion [80,81].

## 6. Curcumin-Loaded Nanoparticles as Therapeutic Agents for Drug Targets

Despite the issue of bioavailability, curcumin is a very popular therapeutic agent, especially when used in conjugation with nanoparticles. The enhanced activity has contributed to its use as a popular carrier molecule where the nanoparticle loaded with curcumin offers better therapeutic effects compared to bare curcumin or pure nanoparticle. Green gum acacia-based silver nanoparticle was synthesized and loaded with curcumin and tested against breast cancer and melanoma. Silver NPs showed good loading capacity when curcumin was used [82]. Curcumin was incorporated into hydrogels and also conjugated with silver nanoparticles to study the release profile. The conjugation helped in the increased release of curcumin by photoactivation, where the selectivity of tumor cells was promoted by photodynamic therapy (PDT). Curcumin was activated by the low-intensity light, and this increased the bioavailability and EPR effect when tested in colorectal cancer cell lines by a process called Metal-Enhanced Singlet Oxygen Generation (MEO) effect [83]. TNBC cells, which, in normal cases, respond to conventional drugs in a minimal mode, were subjected to a novel mode of treatment called electrochemotherapy (ECT), where electroporation with turmeric-conjugated silver nanoparticles (TurNP) was employed. A seven-fold increase in cytotoxicity was observed that was modulated by multiple signaling pathways [84].

PEGylated curcumin conjugated with magnetic nanoparticles served as a vehicle for dual targeting in MCF-7 cell lines. The low toxicity exhibited by the nanocurcumin supports its use as a carrier molecule. However, curcumin served as a therapeutic molecule rather than a vehicle when encapsulated in liposomes and showed enhanced cytotoxicity when treated in colon cancer cells. The acidic conditions facilitated the easy absorption and cytosolic delivery of the nanocurcumin in the colorectal model studied [85]. HCT-116 and MCF-7 cell lines treated with curcumin-gold nanoparticles in such a condition showed reduced viability due to apoptosis induction [86]. Nanocurcumin can overcome the limitations of natural curcumin in terms of stability, bioavailability, and long-term preservation and can act on a wide variety of target molecules ranging from transcription factors, growth regulators, signal transducers, etc. These properties have favored the use of nanoformulations of curcumin over the native form, as the versatility of the nanoformulations can be employed to develop a more suitable and effective therapy. The enhanced solubility and dispersion potentially help in the retained accumulation of nanocurcumin at the site of action resulting in better cytotoxic effects on cancer cells. The passive targeting of nanocurcumin may be influenced by the smaller size and charge around the nanoparticles resulting in the EPR effect [23].

**Figure 7:**
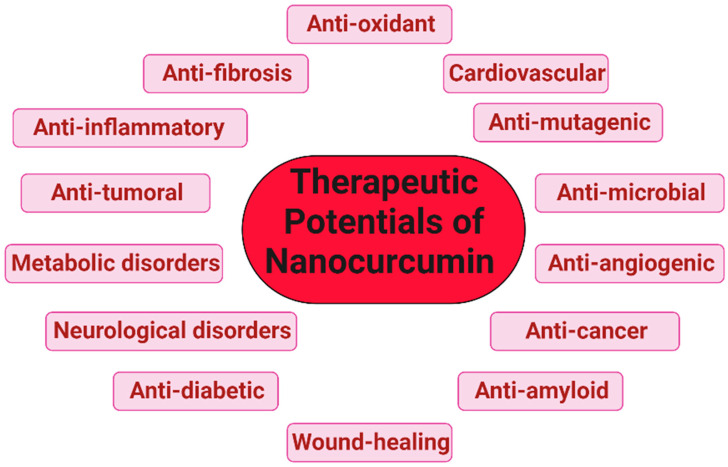
Therapeutic potential of nanocurcumin—the various nanoforms of curcumin have different effects on diverse disease conditions.

Loading curcumin with other compatible carriers and drugs offers a synergistic response where the combined effect may help to increase the toxicity of the conventional drug. The accompanying side effects of conventional drugs can also be minimized with a better payoff in terms of anti-cancer effects. Combinational therapy is almost always beneficial because there is always a second drug loaded with curcumin which already has established, documented evidence for therapeutic effect. The various therapeutic effects of curcumin are enumerated in Figure 7.

## 7. Anti-Cancer Mechanisms of Curcumin

### 7.1. ROS-Mediated Apoptosis Induced by Nanocurcumin

ROS is an important player in the maintenance of homeostasis of the cell. Many critical signaling molecules involving immune responses and growth and differentiation are directly influenced by the levels of various ROS molecules. Stress activates redox signals that, in turn, are found to trigger apoptotic signals mediated by cytochrome C release and caspase activation. Curcumin incorporated with metal oxide nanoparticles was assessed in A549 cells and was found to induce oxidation and ROS generation [87]. The influence of curcumin on ROS was studied earlier, and the pattern of inhibition revealed a major dose-dependent mechanistic interference with protein kinase C (PKC) as well as the regulation of calcium. Increased cytosolic Ca^2+^ influx is known to increase ROS levels. The anti-oxidant and anti-angiogenic properties of curcumin help in the inhibition of Ca^2+^ inflow and also PKC activity [88]. The facile synthesis of nanocurcumin encapsulated with zinc oxide nanoparticles induced apoptosis mediated by ROS, followed by blockage of migration of human lung cancer cells (A549 and HEL-299). Incorporating the zinc oxide nanoparticles favored a pH-responsive activity, resulting in elevated ROS levels and mitochondrial damage [87]. Curcumin complexed with glucose gold nanoparticles (Glu-GNPs) was found to increase the levels of ROS under hypoxia in MCF-7 and MDA MB-231 cell lines. The hypoxic condition induced by curcumin Glu-GNPs inhibited the activities of HIF-1α and the heat shock protein 90 (HSP90). This helped in enhancing the sensitivity of tumor cells to the radiation therapy [89].

ROS behaves as a double-edged sword—it acts as an activator or suppressor of cancer cells based on the tumor microenvironment. In the majority of cancers, ROS is found to be in elevated doses strengthening the observation that ROS favors the cancer growth [90]. The various molecular mechanisms are controlled to induce a higher production of ROS in the tumor cells either by activating the pro-oxidant mechanism or by suppressing the antioxidant system. Balancing ROS in cancer cells is of utmost importance as ROS favors aberrant homeostasis. Since redox signaling and oxidative stress are important events in cancer progression, the effective mode of ROS regulation by curcumin emphasizes the role of curcumin in maintaining homeostasis.

### 7.2. Influence of Curcumin and Nanocurcumin on Various Signaling Pathways

The molecular targets of curcumin and nanocurcumin are so diverse that curcumin is known to physically bind with 33 different proteins and related molecules. The capacity to modulate the molecular and biochemical cascades makes them unique in cancer therapy. The molecular interaction is either direct or happens through the modulation of the expression of genes and gene products. To achieve modulation of gene expression, the signaling pathways are targeted. Some reports also indicate the role of curcumin in targeting the nutrient supply and metabolism of tumor cells as a means of controlling their growth. Glycolysis was suppressed in gastric cancer cells by the enhanced production of ROS and the subsequent stimulation of YAP (Yes-associated protein) and JNK (c-Jun N-terminal kinases) signaling pathways [91]. The signal transducer and activator of transcription 3 (STAT3) is a transcription factor that acts as a novel target for tumor therapy. Many other molecular events are modulated by STAT3, and thus, it forms an ideal target molecule for the anti-tumor action of curcumin. Papillary thyroid cancer was subjected to curcumin treatment that resulted in apoptosis mediated through Jak/STAT3 signaling pathway. Aberrantly activated Janus kinase (JAK) induced the up-regulation of STAT3, contributing to the pathogenesis of thyroid cancer [92]. Curcumin was developed as a novel inhibitor of the STAT3 pathway where treatment with curcumin suppressed the expression of phosphorylated STAT3 in a dose-dependent manner in normal broncho epithelial cells (AALE) and human lung adenocarcinoma cells (H441). This resulted in a reduction in the proliferation of both cell lines as well. By suppressing the STAT3 pathway, immunosuppression of the tumor microenvironment was also achieved.

The Wnt signaling pathway plays an important role in the proliferation and differentiation of cells. Anomalies in the signaling pathway lead to pathological repercussions, including cancer. Down-regulation of the Wnt signaling pathway in tumor cells sensitized with curcumin resulted in apoptosis and cell cycle arrest. The Wnt pathway is blocked by the enhanced expression of microRNA (miRNA)-126a-3p, thus inhibiting osteogenesis and highlighting the collateral damage that curcumin brings about on other organs [93]. Two other intracellular signaling pathways of prime importance in cancer proliferation are the phosphatidylinositol 3-kinase (PI3K)-Akt and the mammalian target of rapamycin (mTOR). These unique, interconnected pathways are actively involved in cell cycle regulation and, thus, serve as a potential target for cancer therapy. Curcumin suppressed the expression of phosphorylated Akt and mTOR proteins in glioblastoma cells (U87), but the expression of tumor suppressor genes PTEN and p53 were promoted [94]. The invasiveness of the glioblastoma was controlled by the suppression of the signaling molecules together with apoptosis induction. This dose-dependent response to curcumin was reported as a significant mode of controlling the growth of those tumors that are aggressive and have poor prognosis.

The polymeric encapsulated form of curcumin induced anti-cancer activity in breast cancer (MDA-MB-231) and lung cancer (A549) cell lines by modulating the expression of hypoxia-inducing factors (HIF) and the p65 subunit (RelA) of the transcription factor, NF-κB. Under hypoxic conditions, administering nanocurcumin resulted in elevated levels of both HIF-1α and p65 (RelA) in the treated cell lines [95]. Membrane-bound receptor tyrosine kinase (RTK) helps in the maintenance of homeostasis, and alerting the activation of RTK will affect the normal processes involved in proliferation, differentiation, migration, etc., contributing to the development of various types of cancers. Inhibitors of RTKs are promising as targeted therapeutic agents as they are involved in several stages of cancer development. There are around 20 classes of RTK in humans, and therefore, there is a range of targets available for therapeutic intervention. Ranging from proliferation to survival, vascular permeability, and migration, many signaling cascade processes are guided by RTKs. Curcumin is found to inhibit almost all components of the tyrosine kinase pathway and the downstream pathway, such as the PI3K/Akt, MAPK, Jak/STAT3, NF-kB, etc. [96,97].

Signaling pathways are undoubtedly one of the major targets in cancer therapy. Curcumin is found to affect all the major, well-characterized signaling pathways, such as NF-κB, MAPK, JAK-STAT, and PI3K-AKT. Molecular alterations in the signaling pathways can act as precursor events for designing new therapeutic molecules for cancer therapy. With the wide range of signaling molecules as molecular targets, curcumin and nanocurcumin can be potentially developed into molecular entities for precision medicine.

### 7.3. Curcumin and Cell Cycle Pathways

The in vitro anti-proliferative effect exhibited by curcumin in human osteosarcoma (CRL 1543) resulted in cell cycle inhibition and apoptosis. Cell cycle arrest was observed in G1/S and subsequently in the G2/M phase. The cell cycle arrest at different phases in the same treated sample was a novelty. The cells treated with curcumin showed decreased Cyclin D1 levels, resulting in the G1/S phase arrest. The G1 to S phase transition of cells is due to Cyclin D1. Cdc2/cyclin B complex, which is responsible for the progression of cells from the G2 to M phase, is sequentially arrested in the cells treated with curcumin which explains the subsequent G2/M phase arrest [98]. G1 and G2/M phase arrest was also observed in HL-60 and K562 cells treated with curcumin [99]. Zein/pectin nanoparticles loaded with curcumin exhibited a significant effect on the cell cycle in human hepatoma cells (HepG2). Cell cycle arrest at S and G2/M was observed in cells treated with encapsulated curcumin [100]. Similar effects were found for curcumin-loaded ZIF-8 nanoparticles coated with hyaluronic acid, which effectively blocked the cell cycle progression at S and G2/M [101]. Curcumin co-functionalized with pectin induced cell cycle arrest, observed as an increase in the cell population at the G2/M phase in SW480 cells. A total of 64.43% of cells were found to be arrested at this phase when treated with the curcumin formulation [102]. A derivative of curcumin and a novel heat shock protein 90 (HSP90) inhibitor, Co86, induced apoptosis and cell cycle arrest in human cervical carcinoma Hela cells. The cell cycle arrest was observed predominantly at the S and G2/M phase, especially during photodynamic therapy, emphasizing the antitumor effect of curcumin in combination therapy. Curcumin served as a source of hydrophobic photosensitizer, a suitable nanodrug delivery system for cancer therapy [103]. However, a combination of curcumin and Metformin inhibited cell growth and induced apoptosis in the prostate cancer cell line (LNCaP) without any effect on cell cycle inhibition. This was confirmed by the expression of the p61 gene that remained unaffected in the experiment [104]. Cells treated with curcumin conjugated with Dox revealed the up-regulation of tumor suppressive gene RB1, and the down-regulation of cyclin-dependent kinase 2 (CDK2) confirmed cell cycle arrest at the G1 phase [20].

Control of cell cycle progression is an evolutionarily conserved mechanism for maintaining the quality and an attempt to control damage to the cell. Many drugs influence cell cycle progression in various ways. Curcumin was also found to contribute to the maintenance of cell homeostasis through cell cycle regulation, as evidenced by the above findings.

### 7.4. Curcumin and Immunomodulation

Modulating tumor immune responses by modifying the complex and heterogeneous tumor immunosuppressive microenvironment is extremely important. The tumor microenvironment contains cells and signaling molecules that help tumor cells to evade the native cell’s immune system by recruiting immunosuppressive cytokines and blocking the surveillance system. Tumor-associated antigens can occasionally produce antigens with enhanced tolerance to the innate immune system, thus controlling the immune system’s function to identify threats. Dietary supplementation of curcumin enhances innate immunity; the addition of curcumin-rich turmeric in the diet bestows better immunity by the modulation of antioxidant-related gene expression [105,106]. Curcumin offers immunomodulatory effects of various nature depending on the tumor microenvironment.

#### 7.4.1. Immunomodulatory Activity of Curcumin and Nanocurcumin

Immunomodulation involves modification of the immune system by suppressing or stimulating specific molecular agents that bring about explicit immune responses where immunity is challenged to fight infections or to target the immune system against any particular disease (such as cancer). To fight such diseases as cancer, the immune system of the patient should be targeted, and the immune response should be shifted toward T helper cells where pro-inflammatory responses are elicited [107]. The immune memory responses studied with curcumin nanoformulation revealed fifteen times more effective results than normal curcumin. This increased immune response contrasts with the immunosuppressive response of bare curcumin, probably due to the increase in secondary humoral antibody levels confirming the ability of nanocurcumin to stimulate the immune system. B-lymphocyte activation is increased, and the antibody-secreting cells become memory cells to antigens [108]. Since memory T-cell responses are harder to study, any instance of altered memory response is positive and will help in understanding how the memory is persisted by the cell after exposure to the antigens.

The immunomodulatory effect of curcumin has been well-known since ancient times. The effect has only increased with the development of nanoformulations of curcumin. Curcumin in nanoform is found to influence the increased release of mediators of immunological response elements in the target site, possibly by the enhanced supply of immunoglobulins.

#### 7.4.2. Role of Curcumin and Nanocurcumin in Immune Tolerance, Co-Stimulation, and Co-Inhibition

Immunosuppression is a hallmark of many types of cancer. Tumor immune tolerance is offered by the overexpression of immunosuppressive mediators, such as programmed cell death-ligand 1 (PD-L1), and by influencing the expression of cytotoxic T cells, natural killer (NK) cells. Curcumin controls the expression pattern of these molecules and offers immune tolerance by preventing tumor cells from escaping the immune checkpoints [109]. The tumor immune microenvironment is regulated by the active participation of the core immunosuppressive pathway mediated by the programmed cell death protein 1(PD-1)/programmed death-ligand 1 (PD-L1) axis. The immune checkpoint is activated when the ligand binds to PD-1 and suppresses anti-tumor immunity. Curcumin reduces the expression of PD-L1 on the surface of tumor cells and inhibits the interaction between PD-1 and PD-L1 with a proportionate increase in the CD8+ T cells. The proliferation of adenocarcinoma cells was inhibited by nanocurcumin, where the T-cell-mediated immune response was activated following a significant reduction in the in vitro secretion of pro-inflammatory mediators from the activated T cells [110]. Similarly, a failure in the Treg (regulatory T cells) suppression of effector T cells can result in immune imbalance because a decrease in the number of Treg cells results in the overactivation of inflammatory cells, and they are a source of infection. Nanocurcumin can restore the frequency and function of Treg lymphocyte cells and the expression patterns of transcription factors, such as forkhead box P3 (FOXP3) and cytokines (TGF-β, IL-10) related to the Treg cells [111,112].

Immune cells rely on a secondary signal called a co-stimulatory signal to activate immune responses in the presence of antigen-presenting cells (APCs). These explicit immune responses are caused by the effective presentation of co-stimulatory signals produced by co-stimulatory ligands on the APCs. Tumor immunotherapy is lately directed against the co-stimulatory and co-inhibitory molecules in the tumor microenvironment. CD86, CD28, ICOS, PD-1, CTLA-4, CD27, CD40, GITR, HVEM, etc., are some of the co-stimulatory signaling molecules affecting the therapeutic effectiveness of any drug [113]. The expression of DC86 was up-regulated by nanocurcumin in dendritic cells (DCs). Wistar male rats (*Rattus norvegicus*) treated with nanocurcumin showed a relatively increased expression of CD4+, CD8+, and CD28+ counts by 96.89, 51.11, and 12.50 %, respectively, as compared to the control group [114]. When the expression of Treg-mediated FOXP3 is decreased, anti-tumor immunity is stimulated [115]. The inhibitory function of Treg is also regulated by the cytotoxic T-lymphocyte-associated protein 4 (CTLA-4) that is constitutively expressed in Tregs [116]. The anti-inflammatory effect of nanocurcumin resulted in an increased expression of Treg and related inflammatory mediators, resulting in reduced inflammation [112]. Similarly, the inducible T-cell co-stimulator (ICOS) is an inducible co-stimulatory molecule found on activated CD4^+^ T cells, sharing structural and functional similarity to CD28. It is an effective activator of the PI3K/Akt signaling pathway. It could be used to identify immunocompetent T cells in solid tumors with a synergistic effect with CTLA-4 blockage. Curcumin can down-regulate CTLA-4, reducing the expression of FOXP3 and the nuclear translocation of the p65 subunit of the nuclear transcription factor, NF-κB [117].

Immunosuppression by the cancer cells makes the patient resistant to therapies. The cells become more progressive and help in further escape from the cell immune machinery. Therefore, it is of utmost importance to identify molecular targets that can effectively modulate the immune suppression exhibited by the cancer cells. It will help in identifying different modes of therapies by targeting the immunosuppressive cells in the tumor microenvironment.

#### 7.4.3. Immune Checkpoint Modulation by Curcumin and Nanocurcumin

Immune checkpoint regulates the immune system of normal cells through ligand-receptor interactions to prevent the indiscriminate destruction of cells by the immune system. However, cancer cells are highly tolerant of the cells’ immune system and can evade the immune checkpoint effectively and efficiently. The immune checkpoint receptor ligands to PD1 and CTLA-4 are up-regulated in cancer cells as part of dodging the immune system, thus silencing the T-cell activation [116]. Interleukins and other stimulatory mediators are blocked along with cell survival regulators. This results in checkpoint inhibition in cancer, thus promoting tumor cell progression. It is, therefore, imperative to develop immune checkpoint blockers in cancer cells to increase the immune response in cancer cells. Curcumin, with a well-known modulatory effect on the expression of PD1 and CTLA-4, can act alongside immune checkpoint blockers to prevent cancer progression [117]. The blocking of immune checkpoints is proving to be a promising approach to controlling tumor cell growth.

In brief, immunomodulation is one of the important modes of action of curcumin and, by default, of nanocurcumin. The immune modulation by curcumin has been exclusively studied with various targets and molecular pathways. The results discussed above are not all-inclusive, but the general trend about the nature of the action of curcumin and its nanoform in immunomodulation is quite evident from these results. The involvement of curcumin in modulating cytokines and other immune response molecules such as Treg and CTLA-4 suggests that curcumin is a potent immunomodulator, especially as it elicits inflammatory responses mediated by pro-inflammatory modulators. The strong evidence of the involvement of curcumin and its nanoform in signaling pathways points out the importance of the compound in immunoregulation. However, there is a drastic gap between experimental data and translational research in this area. Hence, there is a huge possibility for further clinical studies that can strengthen the experimental evidence.

## 8. Application of Various Nanoforms of Curcumin in Different Types of Cancer

The emergence of curcumin-based nanoformulations is reassuring as it can be employed as a subservient drug molecule in cancer therapy. The theranostic applications of nanocurcumin coupled with upconversion nanoparticles (UCNP) confirmed its therapeutic potential. UCNP-PLGA-nanocurcumin was found to be distributed mainly in the liver and lungs. Nanocurcumin in the complex acts as a photosensitizer and an acceptor of the resonant energy transfer through the donor ion Thulium of the UCNP. This process was known to activate ROS and bring cell death in cancer cells [118]. The penetration power of nano-based formulations into the tumor mass is almost always reported as a limitation. However, the UCNP-PLGA-nanocurcumin was found to overcome this limitation in rat C6 Glioma cell tumor spheroids, where the accumulation of the nanoparticles was achieved in one hour with a penetration depth of 100 µM at the least. The nanoformulation of curcumin also improves solubility and stability with better penetration power to deliver the drug to the cancer cells. Curcumin in chitosan-polymeric nanoform functionalized with hyaluronan resulted in the fast release of the drug in HT-29 and CaCO_2_ cell lines with better cellular uptake. CENPs, the polymeric curcumin nanoparticles designed using the cationic copolymer Eudragit E 100, exhibited 19-fold toxicity when tested on C-26 cell lines [119]. Curcumin nanogels, the miniature versions of hydrogels, have a porous framework that will favor drug conjugation and storage. They were administered through a transdermal path for the treatment of skin cancer. These nanogels were effective in inciting toxicity in A375, the human skin melanoma cells.

All these effects of curcumin have favored clinical trials with curcumin as the lead compound or in conjugation with various compounds/drugs. Some of these studies have also entered Phase II trials, highlighting the promising role of curcumin in cancer therapy. The clinical studies on the various nanoforms of curcumin and their effect on different cancers are represented in Table 4.

## 9. Route of Administration of Curcumin and Its Nanoform

Solubility, being a major issue with curcumin, presents a challenge to deliver curcumin to the target cells without losing its therapeutic potential. The route of administration of the drug plays a significant role in the result of the treatment. For the efficient delivery of curcumin to the appropriate target site, different routes are followed. Taking into consideration the various limitations of curcumin, such as limited bioavailability and solubility, non-invasive and invasive methods were followed to obtain the optimum results in treatment using curcumin and its nanoforms.

### 9.1. Non-Invasive Modes of Delivery

Non-invasive methods were employed in ancient times to cure various ailments, such as microbial infections, burns, cuts, wounds, etc. This mode of treatment is the least harmful mode of delivering the drug to the site of action.

#### 9.1.1. Topical Application

Most of the ancient methods of administrating curcumin depend on the topical application of curcumin. This is a typical example of a non-invasive mode of delivery of curcumin. The ancient people in India, especially grandmothers, knew the antiseptic power of curcumin and applied it as a paste to cure wounds, acquire relief from insect bites [1], in cases of psoriasis, etc. [131]. The clinical and microbiological effects of the topical application of curcumin nanoparticles were tested and confirmed in female BALB/c mice against oral candidiasis caused by *Candida albicans*. The number of *Candida* colonies was reduced considerably on this treatment—the effect was comparable to that of the standard antimycotic drug, nystatin [132].

#### 9.1.2. Oral Administration

The most common way of delivering a drug is by oral route as it is the safest and the least intrusive mode of drug delivery. The route is very versatile in including all types of drugs—solid, liquid, capsule, and even powder. Patients prefer the oral route as it is simple and pain-free. However, due to the low solubility and bioavailability of curcumin, the oral administration of curcumin is not as effective as some other traditional drugs. The tissue distribution of curcumin is affected by portal circulation and lymphatic transport. Low solubility interferes with the circulation of blood. However, all these drawbacks are overcome by designing nanoformulations of curcumin where the nanoforms increase the solubility of curcumin and make it available for blood circulation and distribution to various tissues. Immediate degradation upon delivery is prevented, and curcumin is made available for absorption in the liver to enhance the therapeutic effect [133].

#### 9.1.3. Nasal Administration

The nasal administration of drugs is favored when the patient is unable to take the medicine via the oral route, especially when the patient is unconscious and does not have the reflex to ingest the medicine. Micro or nanoemulsions of curcumin can be designed for the intranasal uptake of nanocurcumin. A microemulsion of mucoadhesive curcumin was developed, and the comparative brain uptake of the formulation was studied. Compared to intravenous administration, the nasal route was found to be more promising for targeting curcumin in brain tissues [134].

#### 9.1.4. Pulmonary Route

Local diseases of the respiratory tract have been treated by inhalation therapy from time immemorial. Drugs that are active pharmacologically are delivered through the pulmonary route as an optimal mode of delivery. The thin air–blood barrier, abundant vasculature, and the large surface area of the lungs enable easy absorption of these drugs. Curcumin liposomes, as the dry powder, were sprayed on the lungs of experimental animals for the treatment of primary lung cancer. As the powdered spray reached the site of action directly, the effective biological activity was also found to be enhanced [135]. Recent suggestions for the pulmonary route of curcumin administration are cyclodextrin-formulated nanocurcumin/proliposomes [136].

### 9.2. Invasive Modes of Delivery

The invasive mode of delivery is a means of direct delivery of the drug to the site of action. Encapsulation of curcumin in liposomes and other forms, such as carbon dots, has increased the potential for drug delivery.

#### 9.2.1. Intravenous

The hemocompatibility of drugs is an important factor, as their lack may lead to the rupture of blood cells leading to uncontrollable complexities. Moreover, crossing the blood–brain barrier prevents the drug from reaching the tissues where it is needed. Intravenous (IV) application of the drug will help in overcoming these limitations as the drug enters directly into the systemic circulation. The IV push in the vein initiates an immediate therapeutic effect. The application of intravenous PLGA loaded with curcumin nanoparticles in the tumor model (rat glioma-2 (RG2)) limited the growth of tumor cells [137]. The in vivo pharmacokinetic and therapeutic effects of nanocurcumin were observed to be improved drastically upon intravenous delivery. IV delivery is found to improve the pharmacodynamic and pharmacokinetic properties of drugs to a great extent. Curcumin inhibits many signaling pathways and, thus, influences the outcome of many vital metabolic functions.

#### 9.2.2. Intra-Arterial

The intra-arterial infusion of drugs has great potential for the treatment of stroke, cardiovascular diseases, and neurodegeneration. The tunability of nanoparticles has enabled the encapsulation of drugs for better delivery to arterial walls. The retention capacity of the drug is enhanced by intra-arterial infusion, especially when the drug is encapsulated in nanoparticles.

From the various experimental results, it could be understood that oral administration of curcumin has more acceptance than other modes of administration due to the versatility the ease of this route of administration.

## 10. Conclusions and Future Prospects

The treatment of cancer using curcumin nanoparticles is taking a new direction due to the increased bio-availability and the pleiotropic therapeutic roles that it plays in cancer therapy. The oral administration of curcumin is found to have more acceptance than other modes of administration. The physical and chemical modifications upon the conversion of curcumin to nanoform have resulted in better pharmacokinetic behavior. Though signaling pathways influenced by curcumin and its nanoform in human diseases are studied, a lot more needs to be revealed concerning the dosage and the toxic effects of administering the nanoform of curcumin. The complete therapeutic profile of nanocurcumin is yet to be mapped, and there is a huge potential in this area to develop new pathways, targets, and therapeutic combinations so that a better profile of the molecule is obtained for the further development of drugs for the treatment of various diseases. Combination therapy using the existing conventional drugs combined with nanocurcumin is bound to yield better results. Advanced nanoforms of curcumin, such as niosomes and cubosomes (lyotropic liquid crystalline lipid nanoparticles), can help in minimizing the disadvantages of using curcumin for cancer therapy as these can reduce drug toxicity, encapsulate both hydrophilic and hydrophobic drugs, and protect the carrier drug from physical, chemical, and even biological degradation. This system of drug administration favors topical application, which is an added benefit of using niosomes and cubosomes [138]. These novel forms of nanocarriers can outperform traditional carriers, such as liposomes, due to their transfection and delivery efficiency. This review has touched upon novel aspects of curcumin nanoformulations and the various aspects in which it has contributed to cancer therapy. Much emphasis has been given to immunomodulation by curcumin which is going to be a novel approach in cancer therapy. Various routes of administering curcumin are also discussed in detail. It is expected that this review will pave the way for further research in nanocurcumin and its application in various diseases, including cancer.

## Figures and Tables

**Table 1:** The indigenous practices and curative effects of turmeric.

Indigenous Practice	Curative Effect	Reference
A poultice of turmeric is applied to the perineum	The healing of any lacerations in the birth canal after childbirth	[3]
Powdered turmeric is taken with boiled milk	Cure cough and related respiratory ailments	[3]
Roasted turmeric	Given as an anti-dysenteric for children	[4]
Turmeric paste applied on bruises	Cures bruises and relieves pain	[5]
Turmeric decoction	Relieves - dyspepsia - acidity - indigestion - flatulence - ulcers	[6]
Poultice of turmeric	Applied on - wounds for wound healing - acne to remove acne - insect bite site to relieve pain	[7]

**Table 2:** Clinical trials for the treatment of various types of cancers using curcumin and its nanoform.

ClinicalTrials.gov Identifier	Cancer Type	Phase of Study	Primary Purpose of the Study
NCT03769766	Prostate cancer	Phase 3	Treatment
NCT03980509	Breast cancer	Phase 1	Treatment
NCT01042938	Breast cancer	Phase 2	Treatment
NCT02439385	Colorectal cancer	Phase 2	Treatment
NCT03211104	Prostate cancer	-	Treatment
NCT02724202	Metastatic colon cancer	Early phase 1	Treatment
NCT01160302	Head and neck cancer	Early phase 1	Basic science
NCT03847623	Breast cancer	-	Other
NCT03072992	Advanced breast cancer Metastatic breast cancer	Phase 2	Treatment
NCT01859858	Advanced colorectal cancer	Phase 1	Basic Science
NCT01490996	Colonic cancer metastasis	Phase 1,2	Treatment
NCT01917890	Prostate cancer radiation therapy	-	Supportive care
NCT00192842	Pancreatic cancer	Phase 2	Treatment
NCT03865992	Breast cancer	-	Supportive Care
NCT01740323	Breast cancer	Phase 2	Treatment
NCT02321293	Lung cancer	Phase 1	Interventional
NCT04294836	Cervical cancer, stage IIB	Phase 2	Treatment
NCT00094445	Pancreatic neoplasms, Adenocarcinoma	Phase 2	Treatment
NCT01333917	Colorectal cancer	Phase 1	Interventional
NCT01294072	Colon cancer	Phase 1	Basic Science
NCT00027495	Colorectal cancer	Phase 1	Interventional
NCT02554344	Cervical intraepithelial neoplasia	Early phase 1	Treatment
NCT00295035	Colon neoplasm	Phase 3	Treatment
NCT02724618	Prostate cancer	Phase 2	Supportive Care
NCT02064673	Prostate cancer	Phase 3	Treatment
NCT04731844	Prostate cancer	Phase 2	Treatment
NCT03290417	Prostate cancer	-	Treatment
NCT02598726	Bladder spasm, malignant neoplasm	Phase 1	Treatment
NCT02095717	Prostate cancer	Phase 2	Intervention
NCT01975363	Atypical ductal breast hyperplasia	NA	Intervention
NCT01201694	Advanced cancers	Phase 1	Treatment
NCT02556632	Breast carcinoma	Phase 2	Supportive Care
NCT03192059	Cervical, endometrial and uterine cancers	Phase 2	Treatment
NCT02017353	Endometrial carcinoma	Phase 2	Treatment

**Table 3:** Overview of Inclusion and Exclusion Criteria.

Inclusion Criteria	Exclusion Criteria
Experimental studies	In vivo studies
Publication in English	Other language publications
Full text	Publications with only abstracts
Effect of curcumin/nanocurcumin on cancer cells	Effect of curcumin/nanocurcumin on non-cancerous cells
Detailed data on the mode of therapy	Experiments that need extra data from the author
In vitro and clinical studies	
Functionalized NPs	

**Table 4:** The clinical studies conducted with curcumin with definite outcomes.

Treatment	Type of Cancer	Phase of Study	Number of Patients	Outcome	Ref.
Curcumin, docetaxel	Metastatic breast cancer	Phase I	14	The feasibility, safety, and tolerability of the combination of curcumin and docetaxel therapy were established. Phase II trial in advanced and metastatic breast cancer patients started.	[120]
Curcumin capsules	Colorectal cancer	Phase I	12	Levels of MiG decreased	[121]
Curcumin combined with quercetin	Familial adenomatous polyposis developing into adenocarcinoma	Phase I	5	Polyps decreased after 6 months of combinational therapy. No appreciable toxicity was observed	[122]
Curcumin with piperine	Tropical pancreatitis	Phase I	20	Reduction in the erythrocyte MDA levels with a significant increase in GSH levels	[123]
Curcumin	Pancreatic cancer	Phase II	25	Down-regulation of NF–κB, COX-2, and pSTAT3	[124]
Curcumin in combination with gemcitabine	Pancreatic cancer	Phase I/II	21	Curcumin dose (8 g/day) was observed as above the maximum tolerated dose when taken with gemcitabine with a modest efficacy	[125]
Docetaxel and curcumin	Advanced and metastatic breast cancer	Phase I	14	The maximum tolerable and recommended doses of curcumin were determined in combination with a a standard dose of docetaxel	[120]
Curcumin	Multiple myeloma	Phase I	26	Decreased levels of urinary N-telopeptide of type I collagen Bone turnover Marker	[126]
		Phase I/II	-	Significant down-regulation of the constitutive activation of NF–κB, STAT3, and COX-2 expression	[127]
Turmeric	Lung cancer	Phase I	16	Anti-mutagenic effect	[128]
Turmeric oil and turmeric oleoresin	submucous fibrosis		Groups 1–15 Groups 2–22 Groups 3–21	The potential of turmeric extract against micronuclei formation	[129]
Curcumin	Urinary bladder cancer, arsenic-associated Bowen disease of the skin, uterine cervical intraepithelial neoplasm (CIN), oral leucoplakia, and intestinal metaplasia of the stomach	Phase I	25	Chemopreventive potential of curcumin against cancerous lesions.	[130]

MiG—Mitogen-inducible gene.

## Data Availability

No new data generated during the study.
